# Medical Practice, Urban Politics and Patronage: The London ‘Commonalty’ of Physicians and Surgeons of the 1420s^*^

**DOI:** 10.1093/ehr/cev261

**Published:** 2015-10-29

**Authors:** Justin Colson, Robert Ralley

**Affiliations:** University of Exeter; University of Cambridge

## Abstract

Medical practice in fifteenth-century England is often seen as suffering from the low status and unregulated practice of which Thomas Linacre later complained. Unlike in many European cities, the provision of physic was uncontrolled, and while urban guilds oversaw surgery as a manual art, no comprehensive system of medical organisation or regulation existed. However, in a remarkable episode of the 1420s, a group of university-trained physicians and elite surgeons associated with Humphrey, Duke of Gloucester, briefly established just such a system. While their efforts initially secured approval for a national scheme, it was only in the City of London that they succeeded in implementing their plans. The detailed ordinances of the collegiate ‘commonalty’ they founded provide a unique insight into their attitudes. Drawing on continental models, they attempted to control all medicine within the city by establishing a hierarchy of practitioners, preventing illicit and incompetent practice, and offering treatment to even the poorest Londoners. Yet they failed to appreciate the vested interests of civic politics: achieving these aims meant curtailing the rights of the powerful Grocers and the Barbers, a fact made clear by their adjudication of a case involving two members of the Barbers’ Company, and the Barbers’ subsequent riposte—a mayoral petition that heralded the commonalty’s end. Its founder surgeons went on to revitalise their Surgeons’ Fellowship, which continued independently of the Barbers until a merger in 1540; in contrast, the physicians withdrew from civic affairs, and physic remained entirely unregulated until episcopal licensing was instituted in 1511.

Medical practice in medieval England has long been seen as diverse and largely unregulated, in contrast to continental Europe, where it was frequently tightly controlled by universities (as in cities such as Paris, Bologna and Padua), or by guilds (as in Florence and Valencia). In England there was no unified oversight of medical practice, or of the relationship between physicians, surgeons and apothecaries. While many towns and cities regulated their barbers and surgeons through guilds, physic was subject to no organisational control or minimum qualifications until episcopal licensing for physicians and surgeons was instituted by parliament in 1512. Thomas Linacre was said to have been motivated by the low status of the medical profession, which was ‘engros’d by illiterate monks and Empiricks’, to extend this system of regulation by establishing the College of Physicians of London in 1518. The charter establishing the college explicitly cited the example of Italian cities as its inspiration, contributing to an impression that English medicine had lagged behind continental practice.^1^

The narrative of the absence of regulation in England does not, however, provide the whole picture. There was nothing new in Linacre’s frustration over the contrast between the lack of medical regulation in England and the situation pertaining on the Continent. Long before the early sixteenth-century reforms, English physicians and surgeons already had personal experience of continental models of regulation, acquired through education in continental universities and the French expeditions of the Hundred Years War. Drawing upon this experience, an attempt to implement a scheme for unified medical regulation was proposed in an often forgotten episode of the 1420s. Initially, a proposal to regulate physic throughout England was presented as a petition to parliament by unnamed physicians in 1421. While that proposal was forgotten in parliament, it re-emerged in the form of detailed ordinances for the specific case of the City of London, set out in a second petition by a group of practitioners that included both physicians and surgeons but lacked any members of existing livery companies; those ordinances were approved by London’s Court of Aldermen in 1423. The ordinances set out a comprehensive scheme for defining and regulating medical practice for the City.^2^ They did not, however, clarify the balance of power between physicians and surgeons, between surgeons and other medical practitioners such as barbers and apothecaries, or between royal and urban authorities. This doomed them to failure. The ordinances were only implemented for the brief period of a year; consequently, England would lack medical regulation comparable to that which prevailed elsewhere in Europe for a further hundred years.

Much historical writing on medieval medicine has placed physicians at the top of a tripartite hierarchy of practice, with surgeons below them, and finally apothecaries. The events of the 1420s, however, reveal a very different distribution of power among the different types of medical practitioner, and a different approach taken by the medical professions towards the urban authorities. The proposal enacted in 1423 created a collegiate structure or ‘commonalty’ in which physicians and surgeons had separate but equivalent faculties and were both subject to the same regulations and costs.^3^ The commonalty’s ordinances emphasised not a hierarchy of categories of practice but the educational attainments and proven abilities of its members, who were necessarily limited to an elite minority by high entry fees. Medical practitioners, previously free to engage in both physic and surgery in the absence of regulation, were to be forced to choose one or the other, making the physicians and the surgeons distinct. The commonalty’s officers also had the right of inspection over apothecaries in London, and a duty to ensure that free care was provided for the poor; and its hall was to be the site of lectures and disputations in philosophy and medicine. Despite the implied parity between physicians and surgeons within the commonalty, no place was provided in the ordinances of 1423 for those surgeons who were members of the Barbers’ Company, while it was envisaged that the apothecaries would be regulated, but entirely unrepresented. The effect was to create a distinction between learned and unlearned practice. Ominously for the success of the ordinances, those excluded from the proposed commonalty were precisely those practitioners with existing representation within the civic structures of power.

The events of the 1420s therefore expose wider questions of the balance of power between the royal court and urban authorities, and the attitudes of practitioners towards both of these institutions. Although the petitioners of 1421 possessed impressive court connections—no fewer than four of the petitioners had direct ties to the household of Humphrey, Duke of Gloucester—their proposal was never implemented, despite having been approved in parliament. This was a result of relying upon elite patronage without having any means of putting policy into practice. For similar reasons, while the London scheme of 1423 was implemented, it lasted scarcely a year. Although the mayor and aldermen praised the petition’s aims, the petitioners had failed to consider the implications of their proposals for other London guilds, the members of which they may have considered to be their inferiors, but who nonetheless commanded more influence in civic *Realpolitik* and who were already well versed in the processes of regulation in the City’s courts. The petitioners’ courtly connections held little sway against entrenched civic interests, highlighting the structure of power relations between Crown and City during the fifteenth century.

## I

While the physicians and surgeons of the commonalty envisaged their petition as an attempt to establish a new hierarchy based upon education, they were operating within an environment of strictly defined, and jealously guarded, guild jurisdictions. In London, as in most cities, civic authorities recognised occupational groups defined by the nature of the task they accomplished or the materials they used, not by the status or education of the practitioner. In the early fourteenth century, London’s guilds, or companies, had assumed effective control over entry to the freedom of the City; thereafter the vast majority of citizens became free through completion of an apprenticeship with a recognised company. Consequently, both full economic rights and influence in civic government now depended upon membership of a company. This formal role in civic government encouraged the companies to formalise their administration, and between 1322 and 1396 no fewer than thirty-seven sets of guild ordinances were formally ratified and recorded by the mayor and aldermen. The Surgeons and the Barbers were among this early tranche of companies.^4^

The Surgeons were recognised as a distinct group from at least 1368, when three ‘master surgeons’ were sworn before the mayor and aldermen in the Court of Husting. Although it is not clear whether this implied that a full guild structure was in existence, these individuals were responsible for reporting to the mayor and aldermen the ‘faults of those who undertook cures’.^5^ However, in 1376 the Barbers of London had their guild ordinances ratified, establishing the office of warden, and encompassing regulation of surgery and the ‘cure of maladies’, along with barbery.^6^ The Surgeons and Barbers in London continued in a state of uneasy coexistence until 1540, with masters both of the Surgeons, and of surgery within the Barbers, continuing to be sworn. The Surgeons remained a small group throughout the period, and tried to secure exemption from the city watch in 1492 by emphasising that they had only eight members, while the Barbers had a membership in the hundreds.^7^ In 1409, however, conflict broke out between the companies when the Barbers asked for confirmation of their right to regulate surgery carried out by barbers, as granted in 1376, and appointed two masters of surgery within the Barbers’ Company.^8^ Anonymous complaints were addressed to the mayor in 1415, claiming that ‘some barbers of the said city, who are inexperienced in the art of surgery, do oftentimes take under their care many sick and maimed persons, fraudulently obtaining possession of very many of their goods thereby; by reason whereof, they are oftentimes made to be worse off at their departure than they were at their coming’. However, Mayor Fauconer explicitly endorsed the Barbers’ freedom from supervision by other companies, by ordaining that ‘no barber, practising the art of surgery within the liberty of the said city, should presume in future to take under his care any sick person who is in peril of death or of maiming, unless he should shew the same person, within three days after so taking him under his care, to the Masters [of the Barbers]’.^9^ This judgement regularised the status of those surgeons within the Barbers’ Company, and legitimised direct competition, and almost inevitably conflict, with the Surgeons’ Guild, masters of which continued to be sworn.

At the same time as this jurisdictional conflict was emerging between the Surgeons and the Barbers, several of London’s larger companies were obtaining charters of incorporation in response to increasing royal attempts to regulate corporate activity in the wake of the Peasants’ Revolt. The Skinners and the Grocers were the first in 1394, quickly followed by the Mercers and the Goldsmiths; by the 1420s the majority of London’s larger companies had obtained incorporation via royal letters patent, which often contained new ordinances. However, the disconnection between royal authority and pre-existing civic custom led to a situation where one company’s royal charter could easily conflict with another’s established privileges. Indeed, the overlapping jurisdictions of Crown and City allowed guilds which lacked civic influence to promote their ordinances via the king. This situation led in 1437 to legislation, promoted by the Commons, which required ordinances granted by royal charter to be submitted to the mayor for approval.^10^ Nonetheless, disputes over conflicting grants of regulatory authority continued to provoke quarrels between London’s companies, most notably between the Taylors and Drapers. The Taylors’ charter of February 1440 (which was obtained through the patronage of Duke Humphrey, a member of their fraternity of St John the Baptist) claimed the right to search any premises where cloth was retailed—including those owned by Drapers. As the Drapers’ own searchers were nominally appointed by the mayor, the Taylors’ royal charter was perceived as an attack upon civic privilege.^11^ The Taylors hoped that the election of their only alderman, Ralph Holland, as mayor in 1441 might resolve the situation in their favour; but a draper was chosen instead, provoking members of various artisan companies to resort to violence.^12^

Similar jurisdictional conflicts occurred repeatedly throughout the late medieval period, as those companies lacking civic influence sought to obtain from the Crown rights which encroached upon entrenched customs, only to be defeated within the civic realm. For example, in 1508 the Stockfishmongers broke away from the Fishmongers’ Company and obtained a charter claiming right of search over all fish. Soon afterwards, the mayor ordered the two companies to resolve their differences and merge once more. Similarly, the Haberdashers obtained letters patent granting them the title ‘Merchant Haberdashers’ in 1510, but the City successfully insisted that the title be removed.^13^ Companies spent large sums on lobbying the court and parliament; by this means and through direct ties of patronage they could secure both legislation and letters patent. Yet the writ of the mayor usually overrode that of the king, and currying favour with the mayor and aldermen was much more difficult, as their decisions almost always reflected entrenched civic interests, even those of relatively humble craftsmen.^14^ Exceptions only tended to occur in circumstances where public benefits could be gained without damaging the interests of the dominant interest groups.

The domination of London’s Court of Aldermen and its mayoralty by the ‘great twelve’ companies obliged those companies seeking to expand their rights and privileges to do so outside civic structures, via royal authority. The right of regulation of surgery that was claimed in the ordinances of 1423, however, represented a direct attack on the Barbers. In much the same manner as the Drapers continued to assert their right of search and inspection of the Taylors’ cloth under the authority of their ordinances approved by the mayor and aldermen, so the 1423 ordinances gave the surgeons of the new commonalty the right to judge the work of the surgeons of the Barbers’ Company. The application of the 1421 proposals within the City would have been doomed to failure had the Barbers objected to the selection of Surgeons as the arbiters of licensing, for the royal ordinances would have been regarded as an infringement upon civic privilege. But, by moving to institute their ordinances through the authority of the Mayor, the commonalty initially circumvented opposition from the Barbers.

Despite their shrewdness in thus seeking mayoral approval, the ways in which the commonalty’s elite practitioners, both physicians and surgeons, engaged with the realities of power are illustrative of the limitations of their experience and their outlook. That the petitioners of 1423 chose to deliver their ordinances to the mayor, rather than going through parliament or obtaining a royal charter, showed considerable pragmatism. If we assume that the petitioners of 1423 included those who had (anonymously) petitioned parliament in 1421, or had been involved in that petition’s redrafting in the Lords, then this tactic demonstrates that they had learnt that attempting to enact royal ordinances within the City was ineffective in the face of entrenched privilege. Royal patronage might have been powerful, but the implementation of new policies demanded quite a different set of skills and connections. Indeed, the physicians and surgeons quickly found, as did many other London guilds which tried to extend their privileges, that success depended upon the ability to move between the separate worlds of court and city.

## II

The petition of 1421 was presented in the penultimate parliament of the reign of Henry V, and called for regulation of the practice of physic (and only physic). The petition claimed parity between the ‘three sciences of life’—divinity, physic and law—and it complained that ‘many unconnyng an [*sic*] unapproved in the forsayd Science practiseth ... so that in this Roialme is ev[er]y man, be he nev[er] so lewed, takyng upon hym practyse, y suffred to use hit, to grete harme and slaughtre of many men’. By contrast, it claimed that in other realms, where only ‘connynge men and approved sufficantly y lerned in art, filosofy and fysyk’, were allowed to practise, ‘no man perysh by uncunning’.^15^ The petition proposed a statute allowing only those men able to produce a testimonial of their graduation in the degree of Bachelor or Doctor of Physic from a university to practise. Sheriffs were to be charged with conducting an inquisition as to the credentials of physicians in their area, and those who were not graduates were to be sent to university, or banned from further practice if they refused.

Carefully drafted, and among the earliest bills presented to parliament in Henry’s preferred English, the petition found favour with parliament and the king, who was present for the first time since 1416.^16^ On 2 May 1421 the substance of the petition was granted by the Lords, but with significant changes. Not only physic, but also surgery, was to be regulated: ‘The lords of the king’s council at the time should have the power, by authority of the same parliament, to assign and designate an ordinance and punishment for such people as shall henceforth meddle in and exercise the practice of the said arts and are not skilled or licensed in them’. Those allowed to practise were defined separately, ‘as befits the same arts’, as ‘physicians in the universities, and surgeons among the masters of that art’.^17^ The initiative for the petition may have come from physicians, but the lords in parliament, drawing upon their experience in the French wars, had seen the virtue of applying these regulations also to surgery. They implied parity between the areas of practice by allowing each to judge their own practitioners. While the petitioners were anonymous, there is a strong circumstantial case for identifying the involvement of Humphrey, Duke of Gloucester, in the Lords’ response. Humphrey was one of the ‘triours’ responsible for judging private petitions at the time, and had at least two of the petitioners of 1423—Gilbert Kymer and John Harwe—under his patronage in the following years.^18^

Events overtook the implementation of the petition, and there is no trace of the Lords having actually created the ordinance or statute that they had been authorised to enact by the parliament of May 1421. Only a month after the parliament, Henry embarked for France, remaining there until his unexpected death in August 1422. In these circumstances, it is understandable that medical licensing might have failed to retain the attention of the Lords. While patronage had probably secured initial approval for the proposal, there was subsequently little imperative to ensure its implementation, which in the form described would have added to the workload of the sheriffs. Although enhancing the regulation of medical practitioners had been recognised as a desirable aim, its failure to be implemented reflected the practitioners’ lack of lobbying power in comparison with those such as the landowners and Merchants of the Staple, whose concerns did make the statute book during these troubled years.

On 15 May 1423 a petition was presented to the Mayor and Aldermen of London by three physicians and two surgeons. It set out ten ordinances for a ‘Co*m*i*n*alte’ of physicians and surgeons in London, to be governed by five officers. The five petitioners claimed these offices: Gilbert Kymer was described as ‘Rectour of medicyns in þe Cite of London’, John Somerset and Thomas Southwell as the two ‘Surveiours’ of the ‘faculte’ of physic, and Thomas Morstede and John Harwe as the two Masters of the ‘crafte’ of surgery.^19^ The ordinances were approved as being ‘good and honest and accordyng to open Reason’, but with the proviso that they could be ‘corrected or amended or hoolly ... adnulled’ should the mayor and aldermen deem them ‘vnproffitable or harmefull’.

The ordinances set out the manner in which the commonalty would oversee ‘alle Phisicians and cirugeans withine þe lib*er*tees of london practisyng in Physik or wirkyng in cirurgy’. Candidates wishing to be allowed to practise medicine must be examined and found ‘able þ*er*to’ by the relevant faculty of the commonalty, and were to pay 100*s* to the Chamber of the Guildhall. They were to swear oaths to fulfil their duties properly, to practise ‘well and trewly’, and to report anyone practising badly or without permission. Those found guilty of false practice were to be punished by the mayor by fine, imprisonment, or a temporary or permanent ban from practice. A physician who had attended university could seek admission as a ‘graduate man’ in the faculty of physic by supplying letters of authority to prove his status; his name would be sent to the mayor and he would ‘holde a place as other men don in þe cou*n*seil of Phisicians’, according to his degree and level of experience.

The commonalty was to be divided into three ‘houses’: two devoted to the business of governing physic and surgery respectively, and one for ‘redyng and Disputac*i*ons in Philosophye and in medicyn’. Its five officers were to be elected by its members. The practice of physic would be overseen by two surveyors, chosen annually from among the physicians, and the practice of surgery by two masters chosen annually from among the surgeons; presiding over all of them was to be a Rector of Medicines, a qualified doctor of medicine selected from among the graduate physicians. In an anticipation of later concerns, it was emphasised that officers of the commonalty were to be English-born, further circumscribing an already exclusive group (although it was implied that aliens could join as non-office-holding members).^20^ The rector’s position was primarily to act as an overseer and arbiter: when he was in London, he was to be present at the ‘houses’ within which physic and surgery were governed, but they would proceed as normal when he was absent; he was to ‘be sworne to be indifferent to bothe þe konnynges’ of physic and surgery, and was forbidden from interfering in either without the consent of the surveyors or masters (respectively). The surveyors and masters, for their part, were required to consult him if he was present.

The commonalty’s officers would oversee the examination of candidates wishing to practise, and would (on pain of a 20*s* fine) be available to be consulted by practitioners who encountered difficult medical cases. A physician was to ‘resceive no cure vpon hym Desp*er*ate or Dedly’ without showing it to the rector or one of the surveyors within two or three days, so that it might be ‘comuned with alle þe Co*m*i*n*alte of Phisicians’, while surgeons were to consult over dangerous treatments—especially cutting and cauterisation—with the rector and one of the masters of surgery ‘withinne thre or foure daies þat hit may be comuned with þe discrete parte of Cirurgeans’. In doing this, the commonalty was following the practices of the Barbers’ Company, whose surgeons were already required to show dangerous cases to their wardens.

The ordinances also set out costs and fines. In addition to the entry fee, the petitioners expected that the commonalty would receive an income from fining ‘false’ practitioners and apothecaries: the craft of surgery was to retain half of the income relating to surgery, and the faculty of physic half of the income from physic. At least some of that money was to be used to ensure that anyone ‘nedyng þe practyk of Phisyk or þe wirkyng of Cirurgy’ who had ‘fallen in such pouerte’ that they were unable to pay for treatment would nevertheless receive care. The commonalty’s officers would assign a good physician or surgeon free of charge, and pay the practitioner in question from the organisation’s coffers. Attention was also paid to the interests of patients of modest means: no practitioner was to charge any patient an amount that exceeded either the patient’s ability to pay or the value of the work carried out.

The officers were also to oversee the preparation of medicines, working alongside two assigned apothecaries to check for the sale of ‘false Medicyns’, which were to be thrown away, and their sellers punished by the mayor. Until 1617 the apothecaries of London were members of the larger mercantile Grocers’ Company, as a relic of its original formation from the Pepperers’ and Spicers’ guilds. Although they lacked a distinct guild identity, making their identification in the civic records problematic, the apothecaries’ specialism was explicit, and was officially recognised: for example, in 1471 the mayor William Edward, himself a grocer, called a jury of seventeen apothecaries, along with two physicians, to judge confiscated ‘treacle’.^21^ After the establishment of the College of Physicians in the sixteenth century, many apothecaries were in fact prosecuted for practising physic, suggesting that they believed themselves to be capable not just of dispensing medicines, but also of providing cures. While there is very little evidence of London apothecaries practising in this manner in the fifteenth century, it seems unlikely that they had not attempted to do so prior to the College’s interventions.^22^

Despite the claim of the commonalty to jurisdiction over all practice in surgery within the City, no mention was made of those surgeons who were members of the Barbers’ Company, implying that (at the very least) they would have to submit to the adjudication of the commonalty. As the commonalty made no claims to offer apprenticeships or admission to the civic freedom, its members would have been dependent upon membership of another company for their access to citizenship. This distance from civic customs and conventional guild organisation prefigured the position of the College of Physicians, but also indicates the origins of the scheme in both the petition of 1421 and its continental equivalents, and thereby the courtly status of the petitioners. In particular, the fact that the ordinances usurped the jurisdiction of both the Barbers’ and Grocers’ Companies with regard to the regulation of their own members would prove to be the commonalty’s undoing.

## III

The petitioners’ ambition in 1423 was to institute a system of comprehensive local regulation that was unprecedented in England (although the university at Oxford had claimed oversight of practice within that town since before 1400).^23^ While London’s Barbers and Surgeons, along with similar guilds of Barber-Surgeons in cities including Norwich and Newcastle, regulated their own members’ medical activities, physicians had remained unregulated, and no integrated system of regulation for medicine had been attempted anywhere in England. This left the boundaries between different types of practice and training indistinct.

The petition’s plan for medical regulation would, however, have been familiar to the inhabitants of a number of major cities on the continent. The learned medicine of the late Middle Ages had originated in southern Europe, and that remained its focus: unsurprisingly, therefore, those who sought to improve medical practice in England looked to continental exemplars. A succession of English kings and nobles turned to physicians from abroad. Henry IV had, during his period of exile on the continent, made use of the services of the physicians Louis Recouches and David de Nigarellis de Lucca, who accompanied him on his return to England.^24^ His son Henry V was attended by Peter Dalcobace, Portuguese by birth, while his brother John, Duke of Bedford, was served by Philibert Fournier, a physician from Rouen, and another brother, Humphrey, Duke of Gloucester, employed John de Signorellis of Ferrara.^25^ Richard Courtenay, bishop of Norwich, took Peter of Milan with him to France in 1415, and rather later, Henry VI was treated by James of Milan.^26^ Intriguingly, there is little evidence to suggest that this was also true for surgeons in this period: it was not skill for which patrons looked to continental Europe, but learning.

While influential patrons were attracted to the expertise of French and Italian physicians, some of the practitioners themselves turned to the education available at the great medical schools of France and Italy, in preference to the small-scale faculties at Oxford and Cambridge, which struggled because of a lack of teaching masters and a perception that their medicine was old-fashioned.^27^ The record of admissions to degrees at Oxford, the larger of the two faculties, indicates that only one bachelor of medicine and one doctor of medicine graduated in the year 1449–50.^28^ In contrast, sixty-five medical degrees were awarded at Bologna during the fifteen-year period between 1419 and 1434, and, according to the rolls of the academic year 1378–9, the Parisian medical faculty was admitting between five and eight students per year, and awarding an average of about five doctorates.^29^ For Englishmen looking to study medicine elsewhere, the thriving university at Padua (where we know eight medical degrees were awarded in 1434 alone, and nine in 1450) was a particular favourite: William Hatteclyffe, later physician to Edward IV, went there for his doctorate in medicine after receiving an MA from Cambridge, as did the future Provost of King’s College, Cambridge, John Argentine. Moreover, in the sixteenth century, the influential physicians Thomas Linacre and John Caius both completed their medical education there.^30^ In the French and Italian traditions, in contrast with the situation in England, surgery was often treated as an academic pursuit, explained in books and taught at universities.^31^ Consequently, it was—and has remained—easy to characterise English medicine as backward, ill-organised and isolated from the mainstream of continental learning.

It is therefore scarcely surprising that the more ambitious educated practitioners in England set out to emulate elements of continental medical practice in order to improve their status. The medical schools of Paris, Bologna and Padua controlled both teaching and the regulation of medical practice within those cities; a degree from the university of Paris gave the practitioner the right to treat patients within the city.^32^ Elsewhere, similar levels of control were exercised by guilds rather than by university faculties, notably in Valencia and Florence.^33^ Florence’s medical guild had a group, or ‘college’, of graduates within it.^34^ Milan’s College of Physicians dated back to the 1390s and had many similarities with the London commonalty in terms of its criteria for admission; it also served as a particular inspiration for Linacre’s later establishment of the College of Physicians.^35^ The structures which prevailed in these European cities should not be idealised, but they provided visions of control and regulation that were attractive to elite English practitioners who, without a university in London and with small faculties at Oxford and Cambridge, lacked similar institutions to impose and uphold their view of medical practice.

The importance of medical learning within the commonalty was emphasised by the third house to be included in the planned hall: the place for ‘redying and Disputac*i*ons in Philosophye and in medicyn’. The 1421 petition had assumed that the ideal place for evaluating medical competence was a centre of medical learning, and had accordingly nominated the universities to test the qualifications of anyone found practising without a degree. Moreover the Parisian system was centred on a university, which provided the infrastructure so conspicuously lacking in England.^36^ In contrast, the 1423 petition sought to regulate only medical practice in London, and, in the absence of a university in the city, the commonalty had to establish an alternative forum within which to govern medicine. The courts of physicians and surgeons would provide administrative spaces; the house for lectures and disputations would make the hall a centre for medical learning.

While the ordinances of the commonalty drew much from continental, and especially Italian, models, they also contained some features that were novel, and which incorporated some elements of the traditional London guild structure. The commitment to provide free care for the poor, collectively funded by the commonalty, was novel. While London’s Surgeons had sworn to exercise restraint in charging the poor from at least the 1360s onwards, the scheme for free care had few similarities with the majority of continental schemes of public provision of medicine, or with later pre-Poor Law public health schemes (such as that in Norwich where surgeons were employed at public expense, regardless of whether they were members of that city’s Barber-Surgeons’ company).^37^ In this sense the seemingly generous scheme proposed for London could be interpreted as an effort to assert the practical as well as moral dominance of the commonalty’s elite practitioners, against the potential criticism that others provided a more accessible service. The method of collecting fines, half of which were to be delivered to the City Chamber in an effort to secure civic support for the enforcement of the ordinances, also reflected common practice among London’s companies.^38^ In this way, the proposed structure demonstrated that it drew on a range of sources that neatly encapsulated the variety of experiences, and motives, of the petitioners.

At its heart, however, the petition was intended to create an institutional structure to control the practice of medicine in London. Within the Parisian system, surgeons and apothecaries were expected to carry out what physicians advised, but were themselves barred from prescribing medicines or otherwise taking on parts of the physician’s role.^39^ This structure, which has often been described as the ‘medical hierarchy’, placed a comparatively small group of physicians above a more numerous but lower-status group of surgeons.^40^ For a long time, it was customary to ascribe the division of labour to a series of conciliar decrees of the thirteenth century which forbade clerics from practising surgery because it involved spilling blood; university-trained physicians were clerics, it was argued, so university-trained physicians could not practise surgery. This account is no longer tenable. The decrees in question forbade at most a small proportion of surgical procedures and in any case applied only to those clerics in major orders—never a requirement for students even at the more ecclesiastical universities of northern Europe.^41^ Furthermore, it cannot be assumed that all those described as a ‘physician’ had matriculated at a (northern) university, and non-Christian physicians were clearly present in fifteenth-century England.^42^

Recent work has demonstrated that formal systems of medical control were largely limited to urban centres.^43^ Moreover, achieving and maintaining this degree of control required painstaking work—typically carried out by universities (as at Paris) or by medical guilds (as in Valencia and Florence).^44^ For instance, the system that developed in Paris was the result of a concerted campaign by the medical faculty there, which stated its intention in 1271 to prevent all but physicians from prescribing medicines, and spent the next century issuing petitions to successive popes and the French king pleading for help in preventing illicit practice.^45^ It was only through such repeated efforts, and continual re-emphasis of the rationality and importance of the hierarchy of practitioners, that support was secured for a system the boundaries of which could be policed.^46^ It is therefore clear that the efforts of the guilds and universities were not focused on defending a hierarchy but on establishing one. The Parisian faculty specified the types of practitioner who were to be allowed, and set out to exclude anyone not fitting their categories.^47^ In Paris, as elsewhere, those forbidden to practise included women, whose underrepresentation in contemporary records typically means that they are more visible when being prosecuted.^48^ In short, the depictions provided by the guilds and elite physicians of the medical landscape actually masked a far greater variety of healers.^49^

England’s medical milieu, then, was more fluid and less structured than the statements of particular interest groups might lead us to believe. There are glimpses of this in the language used in the few surviving records of practice from this period. Though in England, as in other countries, physicians and surgeons had been routinely distinguished since at least the fourteenth century, older and less specific labels persisted: references can still be found to practitioners as ‘leche’ or ‘medicus’, both meaning simply ‘healer’.^50^ These descriptions overlapped with the newer terminology: in London in 1417 John Love alias Severell was described as ‘leche’ and ‘surgian’ in a single document, while in 1453 John Gyles was called both ‘leche’ and ‘ffecycyan’; the term ‘medicus’ was applied alike to Thomas Stodeley in the record of his appointment as Master of Surgeons on 7 May 1392, and a Cambridge student called Conrad who was granted permission to graduate as doctor of medicine in 1477–8.^51^

The persistence of these terms partly reflected the presence in England of practitioners who moved freely between physic and surgery—practitioners such as Thomas Fayreford, who, in the first half of the fifteenth century, treated patients in Devon and Somerset for a wide range of problems.^52^ Fayreford had probably studied medicine at the University of Oxford without taking a degree, and he kept a commonplace book in which he marshalled standard scholastic authorities to determine the diagnosis and treatment of various diseases.^53^ Among his list of cures and his own case-studies are clear indications that he performed surgical operations as well as the prognosis, diagnosis and prescription typically considered proper to a physician. One recorded course of treatment involved gargling, injection through the nostrils, anointment of the head, bloodletting under the tongue, Jerusalem pills, purging and a theriac.^54^ He also noted how to recognise and where to find the herb ‘gracia dei’, which further suggests that he was collecting his own simples.^55^ This rare glimpse of medical practice in fifteenth-century England is a salutary warning of the shortcomings of the categories of physician, surgeon and apothecary. There are similar clues in the reading habits of practitioners from this period: Richard Dod, a ‘Barber Sorion’ of London, owned a volume containing treatises on urines, herbs and prognostication for illnesses, while John Crophill, the bailiff for a priory in Essex, treated patients with the help of a manuscript covering topics ranging from urines and diet to herbs and phlebotomy.^56^ Under the commonalty’s proposed system, as at Paris, such interests were to become illicit: a physician should not also be a surgeon, and a surgeon should not attempt to practise physic.

The intention of the London commonalty was to establish the physicians and surgeons as distinct, but largely equal, groupings. The organisation was to be divided into separate but parallel groups, a ‘faculty’ of physic, governing internal medicine, and a ‘craft’ of surgery, governing external medicine, each run from its own ‘house’ within the commonalty’s hall. Rather than an attempt by physicians to limit the activities of surgeons, the ordinances were the work of a handful of elite practitioners of both types who wanted to reserve the government of medicine to themselves. They set the fee for joining the commonalty as 100*s*; this was five times the cost of joining the larger established guilds. In contrast, when the surgeons laid down the ordinances for the Guild of Surgeons which they established in 1435, they demanded only 3*s* 4*d* from apprentices gaining full membership.^57^ The commonalty’s entry fee was (and must surely have been intended as) a major barrier to entry, limiting its members to an exclusive group. The 1423 ordinances therefore reveal a few influential physicians and surgeons trying to establish a hierarchy of medical practice in London that they would oversee, and a centre of medical knowledge and government that they would run.

## IV

Patronage linked the petitioners of 1423 to the most powerful men in England, and to the two opposing factions at court. Humphrey, Duke of Gloucester, as Protector of the Realm, had been a key member of the council that had assented to the 1421 petition. Gilbert Kymer had moved into his service shortly afterwards, becoming involved with the commonalty petition almost immediately. Thomas Southwell, described in the commonalty petition as Bachelor in Medicine, was also affiliated to the duke, later becoming embroiled in a necromancy scandal involving Humphrey’s wife Eleanor Cobham. Another of the surveyors of physic, John Somerset, was at this time in the employ of Thomas Beaufort, Duke of Exeter, guardian of the royal person and the leader of the other powerful faction of Henry VI’s reign.^58^ Somerset later became tutor and physician to the king, and has the distinction of having been the only university-trained physician to have sat in a medieval parliament, when he was elected for Middlesex in 1442.^59^

Thomas Morstede was both the most eminent surgeon of his generation and a highly successful administrator and royal servant, holding offices including King’s Serjeant in 1410 and Searcher of vessels in the Thames, in addition to his military service in France until 1421, including at Agincourt. In 1431 he married Elizabeth, widow of William FitzHarry and daughter of the London mayor and Fishmonger John Mitchell.^60^ Quickly making use of this route to civic patronage, Morstede was elected sheriff in 1436 as a member of his father-in-law’s company. Morstede went on to become the driving force behind the approval of ordinances for a Guild of Surgeons in 1435, of which he was a signatory.^61^ The attribution to him of the translated surgical text *Mesue Englished* (BL Harley 1736) by R.T. Beck is, however, now convincingly discounted.^62^ John Harwe, the other surgeon associated with the 1423 commonalty, was also connected with the Crown, as well as having an established civic career. After having been sworn Master of Surgery for the independent guild of Surgeons in 1429, after the commonalty’s disappearance, Harwe was commissioned in the King’s forces and took six surgeons to Calais for Duke Humphrey’s campaign in 1435; this explains his absence from any involvement in the Surgeons’ ordinances, but confirms the close circle of patronage around the petitioners of 1423.^63^ The petitioners of 1423 were therefore exceptional in possessing an unusual combination of both civic and court connections, which had allowed them to transfer their ordinances from parliament to the City – and which offered them the practical authority to put their plans into action.

## V

In 1424 members of the commonalty arbitrated in their only case: a man called William Forest had accused one of their own founders and two senior members of the Barbers’ Guild of a gross error in treating him.^64^ A wound in Forest’s thumb had been bleeding for nine days when, on the ninth of February 1423/4, he called for help from Simon Rolf, a warden of the Barbers’ Company.^65^ Rolf, unable to staunch the blood, turned to two other surgeons, his fellow Barbers’ warden John Dalton, and John Harwe, formerly a Master of Surgery in the commonalty, perhaps in accordance with the ordinances’ requirement that cases which might lead to ‘deth or mayme’ should be shown to one of the masters within four days.^66^ In a show of co-operation between practitioners of different guilds, the men attended Forest a further seven times, but were forced in the end to cauterise the wound, disfiguring his hand in the process.

Forest’s complaint was taken to the Mayor’s Court, and was phrased in terms of a breach of undertaking. In this respect the civic authorities exercised judgement using the same customary framework as was employed in cases of defective goods and workmanship.^67^ Under the terms of the commonalty’s ordinances a jury was called to adjudicate, composed of physicians and surgeons—though no Barbers, despite the involvement of practitioners affiliated to that company. Possibly because of the importance of the defendants, the committee had one extra physician and two surgeons more than necessary. It was headed by Kymer and included the Surveyors of Physic (Somerset and Southwell) and a Master of Surgery (Morstede), all of whom were sworn in for second terms a few days later; and William Bradwardyne, another royal surgeon who had become Vice-Master of Surgery in the August of that year.^68^ Also on the committee were two surgeons from the commonalty (Henry Assheborne and John Forde); and John Corby, described in the record as a ‘practitioner in physic’ (‘in ph*is*ica practicus’), but also named as a member of the Surgeons’ Guild when its ordinances were approved in 1435.^69^ The surgeons adjudicating were explicitly described as ‘enfranchised in surgery’ implying their connection with the commonalty, in contrast with two of the accused, who were described as ‘admitted as barbers solely for the practice of surgery’. The Barbers’ participation was limited to presentation of evidence by their remaining warden, John Parker.^70^ The eventual decision, given in the Chapter House of the Friars Minor, was that Forest had injured his hand when the moon was ‘combust [i.e. close to the sun] in a bloody sign, namely Aquarius, under a very malevolent constellation’, and that the wound had bled on the ninth of February because the moon was in Gemini; the defendants had made no error.^71^

More was at stake than the reputation of three prominent surgeons. An important subtext was the question of who should judge medical practice, especially when carried out by those who were not members of the commonalty. William Walderne, the mayor who had accepted the commonalty’s ordinances in 1423 and a member of the elite Mercers’ Company, had in so doing accepted the delegation of all medical judgements to the commonalty’s leaders. In their adjudication of Forest’s complaint, those officers explored for the first time their role in judging medical competence. Both parts of the commonalty left their mark on the final verdict: the surgeons could judge the skill of the defendants while the physicians assessed the individual nature of the wound and the importance of the stars in the treatment’s outcome. Such decisions about dangerous surgical procedures required, as the petition had emphasised, the expertise of both surgeons and physicians.

The question of who could judge medical practice was particularly pressing for the Barbers, and the Forest case can only have exacerbated their resentment towards the commonalty over its infringement of their rights. The Barbers’ representatives waited until John Michell, a Fishmonger and the father of Morestede’s future wife, was elected to his first term as Mayor before appealing for help in November 1424.^72^ Characteristically ruling in favour of the established civic interest, Michell determined that the masters of surgery within the Barbers’ Company would again be able to ‘exercise the said faculty as fully as they did in the days of Thomas Fauconer, late mayor, and other mayors, notwithstanding the claim which the Rector and Surveyor of Physicians and the Masters of Surgery now newly impose upon the said barbers by virtue of a certain ordinance made during the mayoralty of William Walderne’.^73^

Michell’s decision to remove the barbers from the jurisdiction of the commonalty had important implications. The mayor’s action rescinded the principle of a hierarchy of skill or education in medical regulation, and restored the unfettered right of self-regulation to the Barbers, as a group entrenched within civic tradition. For the mercantile elite governing the city, the dispute was no different from that between the Drapers and the Taylors already mentioned—one company had attempted to usurp the established jurisdiction of another—and the solution was to restore the status quo. While the physicians and surgeons of the commonalty had initially succeeded by transferring their petition to the City, their connections and influence were still primarily based within the royal court. This patronage counted for very little within the liberty of the City, where the mayor was routinely able to overrule rights given by royal charter. The annual rotation of mayors within the conservative oligarchy of the Court of Aldermen, itself populated overwhelmingly from the ranks of the great twelve companies, meant that innovative policies were often short-lived, and ultimately it was these entrenched companies that held sway. Though the Surgeons’ Fellowship survived the collapse of the wider commonalty, the joint commonalty as a venture to recast medical practice in a manner that reflected medical, rather than civic priorities had failed.

The commonalty’s brief existence was further complicated by the power politics of Henry VI’s minority. In 1424 Duke Humphrey journeyed to Hainaut in the Low Countries, leaving Bishop Henry Beaufort in charge of the royal council.^74^ When Humphrey returned unexpectedly in mid-1425 he found London in uproar against the Bishop, who had, partly fraudulently, taken control of the country’s finances. Beaufort ordered the fortification of the Tower of London against the Duke, and there followed a military stand-off on London Bridge. Bishop Beaufort was joined by men from the shires and claimed the support of John Michell, who was still mayor; Humphrey waited until the day after the installation of Michell’s successor to seek civic aid, eventually returning with three hundred armed men from the City. Further upset was only averted when the Archbishop of Canterbury, Henry Chichele, set about mediating between the two parties. Even so, it was 1426 before a settlement was reached and approved by parliament. The allegiances of the commonalty’s chief members were split down the middle by this feud involving its principal supporters: what had begun as a useful pairing of patrons eventually left the commonalty in an unsustainable position, while its founders, undeterred, mostly went on to bigger and better things. Kymer continued to be Humphrey’s physician, while Morstede and Somerset remained close to the Beauforts, and both were in attendance at Thomas Beaufort’s deathbed.^75^ Somerset retained close links with the civic authorities, who in return for his work in promoting the City’s interests awarded him clothing and an annual income, and admitted a client of his to the freedom of the City.^76^

When Henry VIII eventually instituted medical licensing for both physicians and surgeons in 1512, the need for an established infrastructure of enforcement was recognised, and the church was invested with the practical responsibility for administering it.^77^ In the City of London, however, physicians found episcopal administration insufficient and, assisted by growing civic concerns over public health, moved to establish the College of Physicians led by Linacre.^78^ The similarities between the commonalty of the 1420s and the later College are striking: from the emphasis on university credentials, to the structure of president and elects, which mirrored, but were distinct from, typical London companies. In both cases the similarities with continental, and especially Italian models, were obvious.^79^ While the College of Physicians was established under royal patronage, it had several echoes of the earlier organisation, not least its description in the preamble to the Act of Parliament in 1523 affirming the College’s charter as ‘one Bodie and p[er]petuall Co[mm]i[n]altie or Fellisship of the Facultie of Phisik’.^80^ The College’s advantages over its predecessor were however also obvious: firstly, the principle of medical regulation had been established by a monarch who was sympathetic to the College’s aims and able to ensure their implementation; secondly, a clearer, although still fractious, relationship and demarcation of rights and responsibilities was established between the physicians of the College and London’s existing medical guilds.^81^

The failure of the petitions of 1421 or 1423 to create an enduring structure of medical regulation should be considered in the context of the limited ability of the state, or even the city, to enforce regulation. In late medieval London the implementation of almost all royal legislation was delegated to the civic authorities under the terms of London’s privileges, and the position of sheriff was an elected one within the civic bureaucracy.^82^ The petitioners of 1423 had recognised the need to work within existing structures of power by moving their proposals from parliament to the City of London, where their models, which were themselves exclusively urban, were more directly applicable. However, the physicians and elite surgeons, more accustomed to the networks of patronage surrounding university and court, had access to Duke Humphrey but had little experience, and even less of a power base, in dealing with civic authorities. Consequently, their attempt to enlist civic authority proved to be counter-productive, attracting the ire of powerful vested interests in a way that those experienced in guild life would probably have foreseen. The change of mayor brought a shift in influence, and the Barbers were able to press their claim, restoring their own rights of licensing and assessment. The College of the 1510s succeeded where that of 1423 failed by combining royal patronage with a degree of civic pragmatism. The practical exercise of civic power was dependent upon the cooperation of respective companies or guilds; thus, for example, the inspection of cloth was delegated from the mayor, who possessed the legal right to undertake it, to the Drapers, who had the interest and ability to execute it. There would therefore have been no hope of implementing reform against the will of large and powerful civic factions which had a vested interest in the preservation of the status quo. The failure of the commonalty revealed that royal patronage could be of little use at a time when power was so diffuse.

The failure of the commonalty also had profound implications for London’s spectrum of medical practitioners. The original petition of 1421 had sought to define the role of the physician in England through reference to education, excluding not only fraudulent doctors, but also the great number of practitioners without formal education who straddled the boundaries between physic and surgery. In addition to physicians, Humphrey’s circle included prominent surgeons active in French campaigns, who also regarded themselves as learned and distinct from the majority of practitioners. While it is impossible to establish whose initiative was behind the amended ordinance issued by the lords in response to the petition of 1421, these surgeons, it can be assumed, played a role in encouraging it. The resulting alliance between physicians and elite surgeons led to the drafting of the 1423 ordinances presented to the Mayor’s Court, which combined the mutual interests of the two disciplines in defining a class of learned medicine. The commonalty’s disappearance signalled the abandonment of this goal and of that alliance. It also marked the physicians’ withdrawal from civic affairs. No hierarchy of practitioners was established and the range and variety of London’s medical provision remained unchallenged.

While the physicians effectively avoided any further engagement with civic politics until the next century, the learned surgeons had had much more direct motives for their involvement with the commonalty: their claim to exercise jurisdiction over the surgeons within the Barbers’ Company. The surgeons who had been behind the petition were persistent, and the Guild of Surgeons’ ordinances of 1435 betray a similarity of approach to those of the commonalty, along with several familiar names.^83^ In a sense, therefore, the Surgeons went on to achieve their aim of retaining a visible distinction between learned and unlearned practitioners, for they remained a small, but independent, organisation until their eventual merger with the Barbers in 1540. The reasons for much of the opposition to the attempts to establish a college lay firmly in the conflict between the Surgeons and Barbers. If, therefore, the original petition had concerned only physicians, it might have been more effective as it would have presented little threat to vested interests—if, that is, its proponents had had the experience to lobby the civic authorities independently.

## Footnotes

1 C. Webster, ‘Thomas Linacre and the Foundation of the College of Physicians’, in F. Maddison, M. Pelling and C. Webster, eds., *Essays on the Life and Work of Thomas Linacre, c.1460–1524* (Oxford, 1977), pp. 198–222, at 198, 213.

2 We have included a new transcription of the ordinances as an appendix. The document is in London Metropolitan Archives [hereafter LMA], COL/AD/01/010, City of London Letter-Book K, fos. 6v–7v. It is listed in *Calendar of Letter-Books Preserved Among the Archives of the Corporation of the City of London at the Guildhall* [hereafter *Calendar of Letter-Books*], K: *Temp. Henry VI*, ed. R. Sharpe (London, 1911), p. 11. The petition was previously available in imperfect transcriptions in *Memorials of the Craft of Surgery in England from Materials Collected by John Flint South*, ed. D. Power (London, 1886); *A Book of London English, 1384–1425*, ed. R.W. Chambers and M. Daunt (Oxford, 1967), pp. 108–115; and in R.T. Beck, *The Cutting Edge: Early History of the Surgeons of London* (London, 1974), pp. 63–7.

3 We have followed F.M. Getz’s lead in preferring the participants’ own term ‘commonalty’, expressed as ‘Cominalte’ and other more heavily abbreviated forms in the manuscript, to D’Arcy Power’s coinage ‘The Conjoint College’. The term ‘commonalty’ is used here always to refer to the organisation of physicians and surgeons, rather than the commonalty of the City of London, a usage which was also common. The expression ‘Conjoint College’ is a deliberate reference to London’s College of Physicians, founded a century later, and is potentially misleading in more than one way. Many historians have used it while being apparently unaware of its modern origins, and George Clark goes so far as to discuss the meaning of the word ‘college’ in the fifteenth century. See G. Clark, *A History of the Royal College of Physicians of London*, I (Oxford, 1964), p. 27; F.M. Getz, ‘The Faculty of Medicine before 1500’, in J.I. Catto and T.A.R. Evans, eds., *The History of the University of Oxford*, II: *Late Medieval Oxford* (Oxford, 1992), pp. 373–405. On the choice of the term ‘college’ by the College of Physicians, see Webster, ‘Thomas Linacre’, pp. 209–15.

4 C.M. Barron, *London in the Later Middle Ages: Government and People, 1200–1500* (Oxford, 2004), pp. 204–7.

5
*Calendar of Letter-Books*, G: *1352–1374*, ed. R.R. Sharpe (London, 1905), pp. 236–7; *Memorials of London and London Life, in the XIIIth, XIVth and XVth Centuries: Being a Series of Extracts, Local, Social, and Political, from the Early Archives of the City of London, AD 1276–1419*, ed. H.T. Riley (London, 1868), p. 337.

6
*Calendar of Letter-Books*, H: *1375–1399*, ed. R.R. Sharpe (London, 1907), p. 20; *Memorials of London*, p. 393.

7
*Calendar of Letter-Books*, L: *Edward IV–Henry VII*, ed. R.R. Sharpe (London, 1912), pp. 286–7.

8
*Calendar of Letter-Books*, I: *1400–1422*, ed. R.R. Sharpe (London, 1909), p. 84.

9 LMA, COL/AD/01/009, City of London Letter-Book I, fo. 149, reproduced in full in *Memorials of London*, pp. 606–9.

10 Barron, *London in the Later Middle Ages*, p. 210.

11 Changes to existing rights of correction were frequently portrayed as an attack on the City’s privilege, for example by the Cutlers in a dispute with the Goldsmiths in 1404: M. Davies, ‘Crown, City and Guild in Late Medieval London’, in J.A. Galloway and M. Davies, eds., *London and Beyond: Essays in Honour of Derek Keene* (London, 2012), pp. 241–61, at 260–61.

12 M. Davies and A. Saunders, *The History of the Merchant Taylors’ Company* (Leeds, 2004), pp. 73–7; C.M. Barron, ‘Ralph Holland and the London Radicals, 1438–44’, in C.M. Barron and A.L. Rowse, eds., *A History of the North London Branch of the Historical Association, Together with Essays in Honour of its Golden Jubilee* (London, 1970), pp. 60–80, at 62–5.

13 LMA, COL/CC/01/01/11, City of London Common Council Journal 11, fo. 118v; LMA, COL/CA/01/01/002, City of London Court of Aldermen Repertory 2, fos. 110r, 134v; I.W. Archer, *The History of the Haberdashers’ Company* (Chichester, 1991), p. 17.

14 M. Davies, ‘Lobbying Parliament: The London Companies in the Fifteenth Century’, *Parliamentary History*, xxiii (2008), pp. 136–48; I.W. Archer, ‘The London Lobbies in the Later Sixteenth Century’, *The Historical Journal*, xxxi (1988), pp. 17–44.

15
*Rotuli Parliamentorum ut et Petitiones, et Placita in Parliamento* (6 vols., London, 1783), iv. 158.

16 The significance of physicians, who so clearly favoured Latin, petitioning in English should not be understated, but most likely relates to the use of a skilled intermediary in the drafting of the document. See G. Dodd, ‘The Rise of English, the Decline of French: Supplications to the English Crown, *c*.1420–1450’, *Speculum*, lxxxvi (2011), pp. 117–50, at 131–2.

17 ‘Henry V: May 1421’, in *The Parliament Rolls of Medieval England, 1275–1504*, IX: *Henry V, 1413–1422*, ed. C. Given-Wilson (Woodbridge, 2005), available online at http://www.british-history.ac.uk/no-series/parliament-rolls-medieval/may-1421.

18 These two men are recorded as having accompanied Humphrey on military expeditions on the continent: Kymer in Hainault in March 1424, where he completed a regimen of health for the duke (British Library, MS Sloane 4, fos. 63–104r), and Harwe in 1435 as a surgeon overseeing six others in a force sent to relieve Calais (C.H. Talbot and E.A. Hammond, *The Medical Practitioners in Medieval England: A Biographical Register* [London, 1965], p. 154). Humphrey’s donation of several medical works to the University of Oxford may indicate an interest in the subject; they probably demonstrate the influence of Kymer, who was by that stage Vice Chancellor of the university. See A.C. de la Mare, ‘Manuscripts Given to the University of Oxford by Humphrey, Duke of Gloucester’, *Bodleian Library Record*, xiii, 1 (1988), pp. 30–51, and xiii, 2 (1989), pp. 112–21; H.H.E. Craster, ‘Index to Duke Humphrey’s Gifts to the Old Library of the University in 1439, 1441, and 1444’, *Bodleian Quarterly Record*, i (1914–16), pp. 131–5; H.H.E. Craster, ‘Duke Humphrey’s Gifts’, *Bodleian Quarterly Record*, iii (1920–22), p. 45; note, however, V.L. Bullough, ‘Duke Humphrey and his Medical Collections’, *Renaissance News*, xiv (1961), pp. 87–91.

19 For consistency the form ‘Harwe’, adopted by Talbot and Hammond, has been used, although in the manuscript of the petition, and many other locations, he is referred to as ‘Harowe’. See Talbot and Hammond, *Medical Practitioners in Medieval England*, p. 154; F.M. Getz, ‘Medical Practitioners in Medieval England’, *Social History of Medicine*, iii (1990), p. 266. On Kymer, see F.M. Getz, ‘Kymer, Gilbert (d. 1463)’, *Oxford Dictionary of National Biography* [hereafter *ODNB*]; A.B. Emden, *A Biographical Register of the University of Oxford to AD 1500* (3 vols., Oxford, 1957–9), ii. 1068–9; Talbot and Hammond, *Medical Practitioners in Medieval England*, p. 63; Getz, ‘Medical Practitioners in Medieval England’, pp. 259–60. On Somerset, see C. Rawcliffe, ‘Somerset, John (d. 1454)’, *ODNB*; Emden, *Biographical Register of the University of Oxford*, iii. 1727–8; A.B. Emden, *A Biographical Register of the University of Cambridge to 1500* (Cambridge, 1963), pp. 540–41; Talbot and Hammond, *Medical Practitioners in Medieval England*, p. 184; Getz, ‘Medical Practitioners in Medieval England’, p. 267. On Southwell, see Emden, *Biographical Register of the University of Oxford*, iii. 1734–5; Talbot and Hammond, *Medical Practitioners in Medieval England*, p. 356; Getz, ‘Medical Practitioners in Medieval England’, p. 279. On Morstede, see below.

20 For parallels with the London College of Physicians in terms of the treatment of ‘strangers’, see M. Pelling, *Medical Conflicts in Early Modern London: Patronage, Physicians, and Irregular Practitioners, 1550–1640* (Oxford, 2003), pp. 165–88.

21 In this context ‘treacle’ refers to ‘theriac’, a medicinal compound: J.P. Griffin, ‘Venetian Treacle and the Foundation of Medicines Regulation’, *British Journal of Clinical Pharmacology*, lviii (2004), pp. 317–25; *Calendar of Letter-Books*, L, p. 103.

22 Pelling, *Medical Conflicts in Early Modern London*, pp. 110–11, 119.

23 On Oxford, see V.L. Bullough, ‘Medical Study at Mediaeval Oxford’, *Speculum*, xxxvi (1961), p. 606.

24 On Louis Recouches, see Talbot and Hammond, *Medical Practitioners in Medieval England*, p. 204, and also p. 203, under ‘Lewis, or Louis’, as noted by Getz, ‘Medical Practitioners in Medieval England’, p. 269. On David de Nigarellis de Lucca, see Talbot and Hammond, *Medical Practitioners in Medieval England*, p. 33; Getz, ‘Medical Practitioners in Medieval England’, p. 257.

25 On Peter Dalcobace, see Talbot and Hammond, *Medical Practitioners in Medieval England*, p. 246; Getz, ‘Medical Practitioners in Medieval England’, p. 271. On Philibert Fournier, see Talbot and Hammond, *Medical Practitioners in Medieval England*, p. 253. On John de Signorellis, see ibid., p. 182; Getz, ‘Medical Practitioners in Medieval England’, p. 267.

26 On Peter of Milan, see Talbot and Hammond, *Medical Practitioners in Medieval England*, p. 249; on James of Milan, see ibid., p. 98.

27 For an example highlighting the reputation of Oxford medicine, see the discussion of John Gaddesden’s *Rosa anglica medicinae* in Getz, ‘The Faculty of Medicine before 1500’, pp. 390–91. Oxford’s regulations included provisions for when there was only one regent (allowing him to confer with two or three of his predecessors when judging practitioners, and to dispute once a fortnight rather than weekly): ibid., pp. 383–4. In 1470 William Skelton was granted a grace by Cambridge that relieved him of his teaching duties in compensation for having been delayed the previous year when there had been no regent masters at all: *Grace Book A, Containing the Proctors’ Accounts and Other Records, 1454–88*, ed. S.M. Leathers (Cambridge, 1897), p. 77.

28 The admission to degrees in 1449–50 is in T.A.R. Evans, ‘The Number, Origins and Careers of Scholars’, in Catto and Evans, eds., *Late Medieval Oxford*, p. 494. T.H. Aston has estimated Oxford’s size as around 1,700 students and Cambridge’s as around 1,300: T.H. Aston, ‘Oxford’s Medieval Alumni’, *Past and Present*, no. 74 (1977), pp. 6–7; T.H. Aston, G.D. Duncan and T.A.R. Evans, ‘The medieval alumni of the University of Cambridge’, *Past and Present*, no. 86 (1980), pp. 11–27.

29 N.G. Siraisi, ‘The Faculty of Medicine’, in H. de Ridder-Symoens, ed., *A History of the University in Europe*, I: *Universities in the Middle Ages* (Cambridge, 1992), pp. 372–3; C. O’Boyle, *The Art of Medicine: Medical Teaching at the University of Paris, 1250–1400* (Leiden, 1998), pp. 61–7.

30 For the Padua figures, see Siraisi, ‘Faculty of Medicine’, p. 373. On Hatteclyffe, see R. Horrox, ‘Hatteclyffe, William (d. 1480)’, *ODNB*; Emden, *Biographical Register of the University of Cambridge*, p. 292; Talbot and Hammond, *Medical Practitioners in Medieval England*, p. 398, under ‘William Hattecliffe’; Getz, ‘Medical Practitioners in Medieval England’, p. 281. On Argentine, see P.M. Jones, ‘Argentine, John (*c*.1443–1508)’, *ODNB*; Emden, *Biographical Register of the University of Cambridge*, pp. 15–16; Talbot and Hammond, *Medical Practitioners in Medieval England*, p. 112; Getz, ‘Medical Practitioners in Medieval England’, p. 263. On Linacre, see V. Nutton, ‘Linacre, Thomas (*c*.1460–1524)’, *ODNB*. On Caius, see V. Nutton, ‘Caius, John (1510–1573)’, in *ODNB*.

31 See N.G. Siraisi, *Medieval and Early Renaissance Medicine: An Introduction to Knowledge and Practice* (Chicago, IL, 1990), pp. 63–4 and 178–81. Note, however, Bullough’s contention that a study of books in medieval Oxford suggest a focus on surgery which is not clear from its statutes: Bullough, ‘Medical Study at Mediaeval Oxford’, p. 610.

32 Bullough, ‘Medical Study at Mediaeval Oxford’, p. 611. See P. Kibre, ‘The Faculty of Medicine at Paris, Charlatanism, and Unlicensed Medical Practices in the Later Middle Ages’, *Bulletin of the History of Medicine*, xxvii (1953), pp. 1–20; C. O’Boyle, ‘Surgical Texts and Social Contexts: Physicians and Surgeons in Paris, *c*.1270 to 1430’, in L. García-Ballester, R. French, J. Arrizabalaga and A. Cunningham, eds., *Practical Medicine from Salerno to the Black Death* (Cambridge, 1994), pp. 156–85.

33 For medicine and medical regulation in Florence, see K. Park, *Doctors and Medicine in Early Renaissance Florence* (Princeton, NJ, 1985); for the specific claim that this was a more typical model than the university-centred one, see p. 13. For Valencia, see L. García-Ballester, M.R. McVaugh and A. Rubio-Vela, *Medical Licensing and Learning in Fourteenth-Century Valencia*, *Transactions of the American Philosophical Society*, ixxix, pt. 6 (1989).

34 Park, *Doctors and Medicine*, pp. 38–42.

35 Webster, ‘Thomas Linacre’, pp. 212–18.

36 See P. Kibre, ‘The Faculty of Medicine at Paris’; O’Boyle, ‘Surgical Texts and Social Contexts’, pp. 156–85.

37
*Memorials of London*, p. 337. Some cities, such as Parma and Urbino, employed similar models of regulated or subsidised care by all regulated practitioners, rather than direct payment to individual practitioners. The later London College of Physicians made no such efforts: C. Rawcliffe, *Urban Bodies: Communal Health in Late Medieval English Towns and Cities* (Woodbridge, 2013), pp. 291–3; M. Pelling, *The Common Lot: Sickness, Medical Occupations and the Urban Poor in Early Modern England* (London, 1998), pp. 79–102.

38 M.P. Davies, ‘The Tailors of London and Their Guild, *c*.1300–1500’ (Univ. of Oxford D.Phil. thesis, 1994), p. 94, available online at http://ora.ox.ac.uk/objects/uuid:577c6a65-92cb-4f30-b4fd-e123096dbf43. The later College of Physicians had a similar, albeit short-lived, arrangement, whereby half of the fines collected from irregulars were paid (revealingly) to the King: Pelling, *Medical Conflicts in Early Modern London*, p. 296.

39 O’Boyle, ‘Surgical Texts and Social Contexts’, pp. 163–4.

40 For a modern account of the medical hierarchy, with warnings about its looseness as a model, see Siraisi, *Medieval and Early Renaissance Medicine*, pp. 20–23.

41 Of the fifty-four medical men identified as being associated with Oxford during the fifteenth century, thirty-five are known to have been in major orders, and this proportion is probably inflated because many graduates are known precisely because of their presence in church records: Getz, ‘The Faculty of Medicine before 1500’, pp. 394–5; D.W. Amundsen, ‘Medieval Canon Law on Medical and Surgical Practice by the Clergy’, *Bulletin of the History of Medicine*, lii (1978), pp. 22–44, summarised and supplemented with examples in M.R. McVaugh, *Medicine before the Plague: Practitioners and their Patients in the Crown of Aragon, 1285–1345* (Cambridge, 1993), pp. 72–5.

42 Note for instance Sampson de Mierbeawe, a Jewish physician who in 1409 attended Alice Fitzwaryn, the wife of Richard Whittington: Getz, ‘Medical Practitioners in Medieval England’, p. 277.

43 K. Park, ‘Medical Practice’, in D.C. Lindberg and M.H. Shanks, eds., *The Cambridge History of Medieval Science*, II: *Medieval Science* (Cambridge, 2013), pp. 620–21.

44 On Valencia, see McVaugh, *Medicine before the Plague*, pp. 95–103; on Paris, see O’Boyle, ‘Surgical Texts and Social Contexts’, pp. 163–4; on Florence, see Park, *Doctors and Medicine*, pp. 15–46.

45 Kibre, ‘The Faculty of Medicine at Paris’, pp. 1–20; O’Boyle, ‘Surgical Texts and Social Contexts’, pp. 163–4.

46 O’Boyle, ‘Surgical Texts and Social Contexts’, pp. 173–4, for the papal and royal petitions, and *passim* for the argument that the hierarchy was the result of a concerted strategic campaign during the fourteenth century.

47 This argument is made in O’Boyle, ‘Surgical Texts and Social Contexts’, pp. 163–4.

48 Women were not banned from practice by the 1271 statutes, but were nonetheless prosecuted during the thirteenth century as if they were: M. Green, ‘Women’s Medical Practice and Health Care in Medieval Europe’, *Signs*, xiv (1989), pp. 447–9; for a full account of one such prosecution, see Kibre, ‘The Faculty of Medicine at Paris’, pp. 8–12. On the problems faced in studying medical practice by women in the Middle Ages, see Green, ‘Women’s Medical Practice’, pp. 444–6.

49 K. Park refers to ‘the extraordinary variety of practitioners’, poorly represented by models of practice that prioritise formally trained physicians and surgeons: Park, ‘Medical Practice’, p. 620. F.M. Getz writes that ‘The most distinctive feature of medieval medicine is indeed the variety of people who practiced it’: F.M. Getz, *Medicine in the English Middle Ages* (Princeton, NJ, 1998), p. 5.

50 The *Promptorium Parvulorum*, completed around 1440, gives the Latin word for ‘Lech’ as ‘Medicus’: *The Promptorium Parvulorum: The First English–Latin Dictionary*, ed. A.L. Mayhew (London, 1908), col. 257. On the term ‘medicus’, see McVaugh, *Medicine before the Plague*, p. 40.

51 For Love alias Severell, see *Calendar of the Patent Rolls Preserved in the Public Record Office: Henry V* (2 vols., London, 1910–11), *1416–1422*, pp. 124–5; for John Gyles, see S. Jenks, ‘Medizinische Fachkrafte in England zur Zeit Heinrichs VI (1428/29–1460/61)’, *Sudhoffs Archiv*, lxix (1985), p. 224; for Stodeley’s appointment, see *Calendar of Letter-Books*, H, p. 388. On Conrad, see Talbot and Hammond, *Medical Practitioners in Medieval England*, p. 30; *Grace Book A*, p. 126; Emden, *Biographical Register of the University of Cambridge*, p. 155. Instances of ‘leche’ being used as an occupational description in London also include the will of William Asshton, (d. 1393) (LMA, DL/C/B/004/MS09171/001, fo. 285v), and Thomas Forestier’s remark on the death of ‘our felowe Alban a noble leche’ in a plague treatise of 1485 (Talbot and Hammond, *Medical Practitioners in Medieval England*, p. 343).

52 On Fayreford, see P.M. Jones, ‘Harley MS 2558: A Fifteenth-Century Medical Commonplace Book’, in M.R. Schleissner, ed., *Manuscript Sources of Medieval Medicine: A Book of Essays* (New York, 1995), pp. 35–54; P.M. Jones, ‘Thomas Fayreford: An English Fifteenth-Century Medical Practitioner’, in R. French, J. Arrizabalaga, A. Cunningham and L. García-Ballester, eds., *Medicine from the Black Death to the French Disease* (Aldershot, 1998), pp. 156–83. For the dating of his practice, see Jones, ‘Harley MS 2558’, pp. 41–2.

53 On Fayreford’s putative link to Oxford, see Jones, ‘Harley MS 2558’, pp. 42–3; on the sources of information in his commonplace book, see ibid., pp. 47–51, and Jones, ‘Thomas Fayreford’, pp. 168–9.

54 Jones, ‘Thomas Fayreford’, p. 173.

55 The note is transcribed ibid., p. 161, and discussed ibid., pp. 175–6.

56 On Dod, see R.H. Robbins, ‘Medical Manuscripts in Middle English’, *Speculum*, xlv (1970), p. 410. On Crophill, see J.K. Mustain, ‘A Rural Medical Practitioner in Fifteenth-Century England’, *Bulletin of the History of Medicine*, xlvi (1972), pp. 469–76; Talbot and Hammond, *Medical Practitioners in Medieval England*, p. 138; Getz, ‘Medical Practitioners in Medieval England’, p. 265; P.M. Jones, ‘Crophill, John (*d.* in or after 1485)’, *ODNB*.

57 The ordinances are transcribed in Beck, *Cutting Edge*, p. 132.

58 See, for instance, G.L. Harriss, *Cardinal Beaufort: A Study of Lancastrian Ascendancy and Decline* (Oxford, 1988), pp. 129–30, on the Beaufort family, and R.A. Griffiths, *The Reign of King Henry VI: The Exercise of Royal Authority, 1422–1461* (London, 1981), pp. 83–8, on the factions at court.

59 C. Rawcliffe, ‘A Fifteenth-Century Medicus Politicus: John Somerset, Physician to Henry VI’, in H. Kleineke, ed., *Parliament, Personalities and Power: Papers Presented to Linda S. Clark, The Fifteenth Century*, *X* (Woodbridge, 2011), pp. 97–120.

60 Reginald Sharpe, the editor of the calendars of the Letter-Books, was doubtful that Thomas Morstede the surgeon and Morstede the fishmonger and sheriff were the same person, as John Stow had originally claimed; however, Thomas Morstede stated in his will that his wife was Elizabeth, daughter of John Michell, and also made a bequest for the soul of her earlier husband, William Fitz-Henry: see The National Archives [hereafter TNA], PROB 11/1/191; *Calendar of Letter-Books*, K, p. 11, n. 3. An enfeoffment from Michell, a stockfishmonger, to Morstede (alongside Henry Barton, alderman, and John Carpenter) in 1435, and Michell’s description of his daughter as Elizabeth Morstede in his own will, make it very likely that this Morstede was the same Thomas Morstede, fishmonger, who was elected sheriff in 1436: see *Calendar of Plea and Memoranda Rolls, 1413–1437*, ed. A.H. Thomas (Cambridge, 1943), p. 283, *Calendar of Letter-Books*, K, p. 207; TNA, PROB 11/3/519.

61 Beck, *Cutting Edge*, p. 135. John Stow, *A Survey of London: Reprinted from the Text of 1603*, ed. C.L. Kingsford (Oxford, 1908), p. 315.

62 BL, MS Harley 1736, fos. 2–167 has been accepted by scholars as an original vernacular treatise on surgery, and its authorship has been attributed to Morstede. Lang has shown that there is a Latin source for the manuscript, and that this source can be identified as the work of John Bradmore, a London surgeon of the late fourteenth and early fifteenth centuries: S.J. Lang, ‘John Bradmore and His Book Philomena’, *Social History of Medicine*, v (1992), pp. 121–30; M. Carlin, ‘Morstede, Thomas (d. 1450)’, *ODNB*; Beck, *Cutting Edge*, p. 216.

63 Beck identified Harwe with the mercer, John Harowe, who was a common councilman from 1448 to 1458 and MP for the City in 1450 and 1455. However, Harowe the mercer was only enrolled as an apprentice in 1422, making this connection unlikely: *Calendar of Letter-Books*, K, p. 143, Beck, *Cutting Edge*, pp. 89, 100–102. *Calendar of Patent Rolls Preserved in the Public Record Office: Henry VI* (6 vols. London, 1901–10), *1429–1436*, p. 610; *Calendar of the Close Rolls Preserved in the Public Record Office: Henry VI* (6 vols., London, 1933–47), *1435–1441*, p. 27. For John Harowe’s enrolment as an apprentice mercer in 1422, see *Records of London’s Livery Companies Online; Apprentices and Freemen, 1400–1900,* http://www.londonroll.org/event/?company=mrc&event_id=MCEB1596.

64 LMA, CLA/024/01/02/053, Plea and Memoranda Roll, A52, m. 5. For a summary of the case, see M.T. Walton, ‘The Advisory Jury and Malpractice in 15th Century London: The Case of William Forest’, *Journal of the History of Medicine*, xl (1985), pp. 478–82. The judgement is transcribed with notes in: *Calendar of Plea and Memoranda Rolls, 1413–1437*, pp. 174–5. The case is considered, with earlier and later malpractice cases, in M.T. Walton, ‘Fifteenth Century London Medical Men in Their Social Context’ (Univ. of Chicago Ph.D. thesis, 1979), pp. 150–58.

65 No definitive biographical information can be discerned on Forest himself; however, the only testator of this name in London prior to the 1490s was William Forest, barber of St James Garlickhythe, whose will was enrolled in London’s Commissary Court in 1454: LMA DL/C/B/004/MS09171/005, fo. 134v. If the petitioner was the same man, this would add another dimension to the case as part of the conflict between the Barbers and the Surgeons. On Rolf, see Talbot and Hammond, *Medical Practitioners in Medieval England*, p. 325; Getz, ‘Medical Practitioners in Medieval England’, p. 277.

66 On Dalton, see Talbot and Hammond, *Medical Practitioners in Medieval England*, p. 140; Getz, ‘Medical Practitioners in Medieval England’, p. 265.

67 An action of this kind was formally enacted as a writ of *assumpsit*, but in the London context the customary procedures in use by the Mayor’s Court, and often delegated to the courts of the larger Companies, dispensed with such legal niceties. See F.W. Maitland, *The Forms of Action at Common Law* (Cambridge, 1909), pt. V; P. Wallis, ‘Controlling Commodities: Search and Reconciliation in the Early Modern Livery Companies’, in I.A. Gadd and P. Wallis, eds., *Guilds, Society & Economy in London, 1450–1800* (London, 2002), pp. 85–100.

68 For the swearing-in of these men on 10 October 1424, see *Calendar of Letter-Books*, K, p. 41. Morestede and Bradwardyne were sworn on the 17th of August; see ibid., p. 30. On Bradwardyne, see Talbot and Hammond, *Medical Practitioners in Medieval England*, p. 387, under ‘William Bradwardine’; Getz, ‘Medical Practitioners in Medieval England’, p. 281, under ‘William of Bradwardine’.

69 LMA CLC/L/BB/A/003/MS05244, fo. 23v, transcribed in full in *Memorials of the Craft of Surgery*, pp. 307–20. On Assheborne, see Talbot and Hammond, *Medical Practitioners in Medieval England*, p. 74; on Ford see ibid., p. 144, and also the information under the name ‘Henry Forde’ on p. 422, as pointed out in Getz, ‘Medical Practitioners in Medieval England’, p. 261. On Corby, see Talbot and Hammond, *Medical Practitioners in Medieval England*, p. 136; Getz, ‘Medical Practitioners in Medieval England’, p. 265.

70 On Parker, see Talbot and Hammond, *Medical Practitioners in Medieval England*, p. 175; Getz, ‘Medical Practitioners in Medieval England’, p. 267.

71 The committee found that the wounding had occurred ‘luna existente combusta in signo sanguineo vide*lice*t | Aquario sub constellac*i*o*n*e plurimu*m* maliuola’: LMA, CLA/024/01/02/053, m. 5.

72 He was often confused by scribes with William Michell, a close relative and a prominent grocer. On the scribal confusion of William and John Michell see A.B. Beaven, ‘John Michell, Mayor of London’, *Notes and Queries*, 10th ser., xii (1909), pp. 361–2; TNA, PROB 11/3, fo. 232v.

73
*Calendar of Letter-Books*, K, pp. 36–7.

74 For details of this episode, see Harriss, *Cardinal Beaufort*, pp. 134–49; Griffiths, *Reign of King Henry VI*, pp. 70–81; K.H. Vickers, *Humphrey of Gloucester: A Biography* (London, 1907), pp. 170–87; S. Saygin, *Humphrey, Duke of Gloucester (1390–1447) and the Italian Humanists* (Leiden, 2002), pp. 30–47.

75 Rawcliffe, ‘Somerset, John’.

76 LMA, COL/CA/01/01/004, City of London Common Council Journal, 1444–48, fos. 84r, 90v, 144r (the last for admission of Somerset’s client to freedom of the city). See also comments in C. Rawcliffe, ‘More Than a Bedside Manner: The Political Status of the Late Medieval Court Physician’ in C. Richmond and E. Scarff, eds., *St George’s Chapel, Windsor, in the Late Middle Ages* (Leeds, 2001), pp. 75–6.

77 3 Hen. VIII, c. 11: *The Statutes of the Realm*, ed. J. Raithby (11 vols. in 12, London, 1810–28) iii. 31–2.

78 J.H. Raach, ‘English Medical Licensing in the Early Seventeenth Century’, *Yale Journal of Biology and Medicine*, xvi (1944), pp. 267–88, at 271–9; R.S. Roberts, ‘The Personnel and Practice of Medicine in Tudor and Stuart England, 2: London’, *Medical History*, viii (1964), pp. 217–34, at 217–22.

79 Webster, ‘Thomas Linacre’, pp. 212–19.

80 14 & 15 Hen. VIII, c. 5: *Statutes of the Realm*, iii. 213.

81 Pelling, *Medical Conflicts in Early Modern London*, pp. 150–55.

82 Barron, *London in the Later Middle Ages*, pp. 37–42.

83 Beck, *Cutting Edge*, pp. 130–35.

## Appendix: Petition of the Cominalte of Physicians and Surgeons of London

London Metropolitan Archives, COL/AD/01/010, fos. 6v–7v

Conventions used in the transcription:

| for line-breaks, || for page-breaks

[] represents deletions

\/ represents insertions

u, v, i and j, þ transcribed as in the manuscript

ff transcribed as F

expansions italicised

larger text emboldened

The ordena*u*nce and Articles | of phisicions withinne þ^e^ | Cite of London and Sur|geons of þ^e^ same Cite

**The** xv Day of maij þe yere of kyng Henry þe sixte after þe conquest þe first Maister Gilbert | Kymer maistre of art Doctour of medicyns And Rectour of medicyns in þe cite of london Maistre | Iohn Suntbreshete Comensour in Medicyns And maister Thomas Southwell Bachiler in Medicyns þe two | Surveiours of þe facutie of Phisyk in þe same Cite And Thomas Morstede and Iohn Harowe þe two | Maistres of þe crafte of cirurgy with alle þe cirurgeans wirkyng in þe crafte of cirurgy withinne | London comen here and putten vp to þe maire and Aldermen a bille or a petic*i*on in Englissh contenyng | þe honeste of þe Faculte of Phisyk and þe honeste of þe crafte of cirurge and þe co*mun*e proffit of þe cite | in these wordes

**Noble** Lordes for as moche þat þe glorious konnyng of Phisyk and þe crafte of Cirurgy er fro Day to Day | gretlich Disclaundred and sorofully skorned and grete parte of þe peple spillide be wrecched and p*re*sumptuous | practisours in phisyk nought knowyng þe treuthe or ground of þat Faculte of phisyk And be vnkonnynge wirkers | in Cirurgy nought knowyng þe trewe crafte of cirurgy like it to your lordships for þe disclaundre of so high | a Faculte of Phisyk and so worthy a Crafte of cirurgy to be putte awey And that mankynde be nought begiled | from hennesforward be þe Disceites of vnkonnynge practisours in phisyk and vnkonnynge wirkers in þe crafte | of Cirurgy withinne þe boundes of your Fraunches to stable þordinau*n*ce vnderwriten eu*er*more to be obserued.

**In þe first** please it you to ordeyne þat [f] whennesforward alle Phisicians and cirurgeans withinne þe lib*er*tees of london | practisyng in Physik or wirkyng in cirurgy as oon Co*m*i*n*alte be oon Rectour of Medicyns and two Surveiours | of þe Faculte of Phisyk and two Maistres of þe crafte of Cirurgye mowe be gou*er*ned in mane*r* and fourme | suynge That is to say that oon place be hadde withinne þe cite of London contenyng atte lest thre howses seu*er*all | wherof oon be chaired and desked for redyng and Disputac*i*ons in Philosophye and in medicyn And that other | for congregac*i*ons elect*i*ons and counseils of alle phisicians practisyng in phisyk for alle man*er* of articles to be | decided oonly p*er*tenynge to þe Faculte of Phisyk And þe thrid for congregac*i*ons elect*i*ons and counseils of | alle cirurgeans wirkyng in þe crafte of cirurgy for all man*er* of articles to be dealed oonly p*er*tenynge to þe | crafte of cirurgye So þat þe Rectour of medicyns be at bothe if he be p*re*sent in towne as p*re*sident and Rewler | And if he be nat p*re*sent þan þe Faculte of Phisyk and þe Crafte of Cirurgy p*ro*cede as he were present eche | by hym selfe oonly in alle maner of poyntes þat longeth to her konnynges

**A**lso please you to ordeyne þat þe said Co*m*i*n*alte of alle Phisicians and cirurgeans of London eu*er*eche yere of hem | selfe mowe chose and presente to þe Mair of the cite for þe tyme beynge oon Rectour for þe Faculte of | Medicyn by hym to be rewled And þe saide phisicians eu*er*eche yere of hem self mowe chose and p*re*sente be|fore þe mare of the cite of london for þe tyme beyng two Surveiours for þe Faculte of Phisyk to be gou*er*ned | And þe cirugeans of london eu*er*eche yere of hem self mowe chose and p*re*sente to þe Mair of the Cite of | london for þe tyme beyng two Maistres for þe Crafte of Cirurgy to be gou*er*ned by Alwey excepte þat none | be chose Rectour of Medicyns bot he be Doctour of Medicyns maistre of arte and Philosophie or a bachiler in Medi|cyns of long tyme in vertu and konnyng approued if eny suche may be found And if non suche may be found ne | be p*re*sent þan þe Faculte of Phisyk be gou*er*ned oonly by þe Surveiours of þe same Faculte. And þe Crafte of | cirurgy by þe two Maistres of þe same crafte eche by hym self in to tyme þat suche a Doctour may be found | or a Bachiler So þat þe Bachiler ne occupie þoffice of þe Rectour lenger þen suche a doctour of þe condic*i*ons | afore rehersed may be founde Also excepte þat non be chose Rectour ne Surveiours of Phisike ne Maistres | of Cirurgye bot þei be born withinne þe Roiaume of England wisest ablest and most discrete of long | tyme also in vertu and konnyng yproued.

**Also** please it you to ordeyne that no persone withinne þe liberte of þe Cite of London p*re*sume in eny wise to p*r*actise | in Phisyk olesse þan he be examined and found able þ*er*to be þe Rectour and two Surveiours of Phisyk and þe holer | partie of þat Faculte And þan admitted be þe Mair and Aldermen on peyne of C S to paie to þe Chambre | of Gyldhalle for þe co*mun*e proffit of þe Cite no þat no p*er*sone withinne þe saide liberte of London p*re*sume to wyrke | in þe Crafte of Cirurgie olesse þan he be examined and found able þ*er*to be þe seide Rectour in Medicyns and | two maistres of þe Crafte of Cirurgie And þe more and holer p*ar*tie of þe same Crafte And þan admitted | be þe Mair and Aldermen on þe peyne before rehersed paiable in þe same maner ||

**Also** please it you to ordeyne þat no Phisician withinne þe Fraunchise of London resceive no cure vpon hym | Desp*er*ate or Dedly bot he shewe it with alle þe circu*m*stance to þe Rectour of Medicyns or to oon of þe Surveiours of | Phisyk withinne two or three dayes þat it may for þe remedy þerof if eny be possible be comuned with alle þe | Co*m*i*n*alte of Phisicians ne do no þing be way of Medicyne to no paciente by þe whiche it is like to hym or doubte | þat þe paciente myght stande in p*er*elle. Also þat no Cirurgean withinne þe boundes of London resceive no cure | in to his hande of þe whiche may folowe deth or mayme without þat he shewe it to þe Rectour of Medicyns if | he be p*re*sent in towne and to oon of þe two maistres of cirurgy withinne thre or foure daies þat hit may | be comuned with þe discrete parte of Cirurgeans nor he shal nat make eny kutyng or cauterizac*i*on wherof may | folowe deth or mayme without þat he shewe it afore to þe Rectour of Medicyns if he be p*re*sent in towne And to | þe two Maistres of Cirurgy þat it may be comuned with þe discrete p*ar*te of Cirurgeans for saluac*i*on of þe paci|ente and worship of þe Crafte of Cirurgy with that that Rectour Surveiours and Maistres aforeseid be alwey | redy when þei be required to ouerse and decide þe matters aforeseid without eny thing takyng for her labour on | peyne of xx^s^ Alwey obserued þat þe Rectour of Medicyns gif no dome in eny case of cirurgie w*i*t*h*out consent | of þe two Maistres of Cirurgie or of oon of hem with þe discrete p*ar*tie of þe Co*m*i*n*alte of þe Crafte of Cirurgy|ans nor he shal nat make non ordeyna*u*nce nor no constituc*i*ons þat p*er*teneth to þe Crafte of cirurgy withoute | þe consent of þe two Maistres of cirurgy or of oon of hem and þe discretour p*ar*tie of Cirurgeans And | nether of þe two Maistres of þe Crafte of Cirurgy Do þe same with out hym and he be p*re*sent And þe same | article be obserued in þe Faculte of Phisyk

**Also** please it you to ordeyne and stable þat if eny Phisician before þe Rectour of medicyns and þe two Surveio*u*rs | of Phisyk trewly and lawfully be conuicte of false practike in Phisyk or of any other open defawte disclaundred | and worthy accusac*i*on by two or thre trewe men, this Doon anone relac*i*on þ*er*of made to þe mair of the cite of london | he be punysshed by þe saide Mair without delay with peyne pecunier or prison or puttyng out fro*m* alle practyk | in Phisyk for a tyme or for euer more after þe quantite and qualite of his trespas as to þe Mair and Aldermen | by examinac*i*on of þe trouthe and informac*i*on of þe Rectour and Surveiours of Phisyk may be found Also if | eny Cirurgean before þe Rectour of medicyns and þe maistres of cirurgy trewly and lawfully be conuicte | of false wirkyng in cirurgye or of eny other open defawte disclaundred and worthy accusac*i*on by two or thre | trewe men this doon anone relac*i*on þerof made to þe Maire of þe cite of london he be punisshed be þe | mair without delay with peyne pecunier or prison or puttyng out fro*m* alle oþer wirkyng in þe Crafte of | Cirurgy for a tyme or for euermore after þe quantite and qualite of his trespas as to þe Mair and Aldermen | by examinac*i*on of þe trouthe and informac*i*on of þe Rectour and þe two maistres of Cirurgye may be found.

**Also** please it you to ordeyne þat eu*er*eche seke man nedyng þe practyk of Phisyk or þe wirkyng of Cirurgy fallen | in such pouerte þat he sufficeth nat to make good for þe labours of his Phisician or of his Cirurgean if þe cause |pertenynge to physik if he will pleyne hym to þe Rectour of Medicyns or to oon of þe Surveiours of Phisik a gode | practisour shal be assigned by oon of hem besily to take hede to hym without eny expense resceivyng for his labour | vpon resonable peyne to be sette by þe counsell of Phisyk And if it be a cause of Cirurgy if he will pleyne hym to | þe Rectour of Medicyns or to oon of þe two maistres of Cirurgy a good wirker of þe same crafte shal be assigned | by oon of hem besily to take hede to hym without eny expense resceivyng for his labour vpon resonable peyne to be sette | by the Rectour and þe two Maistres of cirurgy and þe holer p*ar*tie of þe same Alwey outake þat none of þe Phisicians | ne Cirurgeans take ou*er*moche mone, or vnresonabely of eny seke bot after þe power of þe seke man and mesurabely | after þe deseruyng of his labour

**Also** please it you to ordeyne þat if eny false Medicyns or sophisticate or made vntrewly be found to selle be þe Rectour | of Medicyns and þe two Surveiours of Phisyk and þe two Maisters of Cirurgy and two Apotecharyes assigned þ*er*to in | þe Shoppe of eny Apotechary or elles where withinne þe boundes of london in eu*er*eche ou*er*seynge of her medicyns þat | þei be demed alwey to be caste a wey by þe Rectour and þe six p*er*sones afore rehersed. And þe Apotechary or other | seller holdyng with hym suche medicyns be punysshed by þe Mair As to þe mair and Aldermen be examinac*i*on | of þe trouthe and informac*i*on of þe Rectour and six p*er*soens afore rehersed may be found ||

**Also** please it you to ordeyne that non be admitted at london for a graduate man in þe Faculte of Medicyns in to þe Comu|nalte of Phisicians bot he bryng before þe Rectour and two Surveiours of Phisyk lettres of Recorde of sufficiente | auctorite or other sufficient witnesse of his graduac*i*on And þanne nedefull þinges don asked before þadmission | he be admitted to practise And after þe worthynes of his Degre and tyme of admission holde a place as other | men don in þe cou*n*seil of Phisicians So þat þe names of eu*er*eche admitted for a graduate man be sent be þe saide | Rectour to þe mair writen

**Also** please it you to ordeyne that þe Rectour of Medicyns of london and bothe þe Surveiours of the Faculte of | Phisyk and bothe Maistres of þe Crafte of cirurgye in þe tyme of þair admission be bound to swere þat in as | moche as in þaim is alle and eu*er*eche constituc*i*ons to þair office belongynge shal obserue or Do to be obserued alle | loue hate fauour and negligence lefte as god and þe holy gospels þaim shall helpe And þe Rectour be sworne to be | indifferent to bothe þe konnynges And also þ*a*t euereche Phisician in his admission to þe practys of Phisyk in london | before þe mair of þe cite in Guyhall shall swere þat he shall practise in phisyk well and trewly not in gevyng | wityngly noious medicyns to eny man nor consentyng to þe yeuer ne he shal entermete of eny sekenes after his trewe | estimac*i*on vnknowyng to hym in eny maner nor in medicyns doyng or makyng he shal not vse eny sigilyng eny wyle | sophisticac*i*on or vntrowthe \so/ god hym helpe and þe holy Eua*u*ngeiles And if he knowe eny man vse eny vntrouthe | of þe forseide malices or nat admitted to þe practise of Phisyk withinne þe boundes of london / hym that he knoweth | so to practise in phisyk he shalle shewe without taryinge to þe Rectour of Medicyns and to þe two Surveiours of Phisyk and her counseill in alle maner of causes lawfall and honest to her konnynge p*er*tenynge | And euereche cirurgean in his admission to þe crafte of cirurgy in london, before þe mair in þe Guyhalle shalle | swere þat he shalle wirke in þe crafte of cirurgye without fraude welle and trewly nat in yevyng layng or vsyng | eny noious medicyns to þe crafte of cirurgye p*er*tenynge nor he shall nat entermete of sekenes sore or hurte pertenynge | to þe Crafte of Cirurgy after his trewe estimac*i*on vnknowynge to hym in eny maner. nor he shall nat vse eny sigi|lyng eny wile sophisticac*i*on or vntrouthe so god hym helpe and þe holy gospels And if he knowe eny p*er*sone vse | eny vntrouthe of þe forseid malices or nought admitted to þe crafte of cirurgy withinne þe boundes of london | hym þat he knoweth so to wirke in cirurgye he shalle shewe without tarynge to þe Rectour of Medicyns and to | þe two maistres of þe crafte of cirurgye and to her counseil And he shalle appere without eny yensaynge atte | callynge of þe Rectour of Medicyns and of þe two maistres of þe crafte of cirurgy and her counseill in alle | maner of causes lawfull and honest to her crafte p*er*tenynge Sauynge alwey priuileges statutes and custumes | of þe cite of london co*m*endably vsed

**Also** please it you to ordeyne þat þe halvendele of þe Mone comynge of þe peynes þat ben rered be a Sergeau*n*t | þ*er*to ordeyned for forfetes made in þe Faculte of Phisyk tourne halvendele to þe chambre [of þe cite] of þe cite of | london And þat other halvendele to þe Faculte of Phisyk as best semeth to þe Rectour and to þe two Surveio*ur*s | of phisyk and to her co*m*i*n*alte to be don And halvendele of þe mone comynge of þe peynes þ*a*t ben rerid by þe Serge|ant aforeseid for forfetes made in þe Crafte of Cirurgye tourne halvendele to þe chambre of þe cite of london | and þat other halvendele to þe crafte of Cirurgy as it best semeth to þe Rectour and to þe two Maistres of Cirurgy | and her Co*m*i*n*alte to be don

**T**he whiche bille after þat hit was redde herde and diligently vnderstande by þe mair and Aldermen for as | mochel as all and eu*er*eche the articles contened þ*er*einne semed good and honest and acordyng to open Reason Therfor*e* | it is graunted by þe forsaid mair and Aldermen þat þe forseid articles from hennesforward be holde stedefastly | and be kept without variau*n*ce and putte to execuc*i*on Outake alwey þ*a*t if eny tyme to come hit seme here to þis | courte eny article aforseide to be vnproffitable or harmefull So þat in alle or in p*ar*celle it nedeth to be corrected | or amended or hoolly to be adnulled / be þe discrec*i*on of þe mair and Aldermen for þe tyme beyng / be hit lefull | to hem þat same article by way of correcc*i*on to adde or take awey þ*er*fro or alle to putte away as hit to hem most | nedfull and spedefull semeth *et*c

